# Resource capture and competitive ability of non-pathogenic *Pseudogymnoascus* spp. and *P*. *destructans*, the cause of white-nose syndrome in bats

**DOI:** 10.1371/journal.pone.0178968

**Published:** 2017-06-15

**Authors:** Michael B. Wilson, Benjamin W. Held, Amanda H. Freiborg, Robert A. Blanchette, Christine E. Salomon

**Affiliations:** 1 Center for Drug Design, University of Minnesota, Minneapolis, Minnesota, United States of America; 2 Department of Plant Pathology, University of Minnesota, Saint Paul, Minnesota, United States of America; CSIRO, AUSTRALIA

## Abstract

White-nose syndrome (WNS) is a devastating fungal disease that has been causing the mass mortality of hibernating bats in North America since 2006 and is caused by the psychrophilic dermatophyte *Pseudogymnoascus destructans*. Infected bats shed conidia into hibernaculum sediments and surfaces, but it is unknown if *P*. *destructans* can form stable, reproductive populations outside its bat hosts. Previous studies have found non-pathogenic *Pseudogymnoascus* in bat hibernacula, and these fungi may provide insight into the natural history of *P*. *destructans*. We compared the relatedness, resource capture, and competitive ability of non-pathogenic *Pseudogymnoascus* isolates with *P*. *destructans* to determine if they have similar adaptations for survival in hibernacula sediment. All non-pathogenic *Pseudogymnoascus* isolates grew faster, utilized a broader range of substrates with higher efficiency, and were generally more resistant to antifungals compared to *P*. *destructans*. All isolates also showed the ability to displace *P*. *destructans* in co-culture assays, but only some produced extractible antifungal metabolites. These results suggest that *P*. *destructans* would perform poorly in the same environmental niche as non-pathogenic *Pseudogymnoascus*, and must have an alternative saprophytic survival strategy if it establishes active populations in hibernaculum sediment and non-host surfaces.

## Introduction

White-nose syndrome (WNS) is an invasive mycosis of hibernating bats caused by the psychrophilic fungus *Pseudogymnoascus destructans*, formerly *Geomyces destructans* [1–3]. *P*. *destructans* was completely unknown before the mass mortality of North American bats began in 2006, but is now recognized as an invasive pathogen from Europe [4–6]. While *P*. *destructans* does not cause mass mortality in its native range [5], up to 6.7 million North American bats died of WNS by 2012 [7] with the possibility of widespread or local extinctions in the future [8,9]. At least seven North American bat species are affected by WNS throughout 29 states and five Canadian providences, including two endangered and one threatened species [10,11]. Even though WNS is now wide-spread, *P*. *destructans* is of a single clonal genotype in North America [12,13]. WNS is an important national economic issue because bats are estimated to perform $22.9 billions worth of agricultural pest control in the U.S. annually [14].

WNS-positive bats shed infectious conidia into the hibernaculum environment where they can persist [15], but it is unclear if *P*. *destructans* can form stable, reproductive populations in sediment and surfaces that can act as permanent infectious reservoirs. Understanding the natural history of *P*. *destructans* on hibernaculum surfaces is critical for WNS management because it affects the feasibility of some intervention strategies. Bats in hibernacula with environmental populations of *P*. *destructans* would be at constant risk of infection regardless of individual treatment, culling, or reintroduction after population collapse.

Since the emergence of WNS, researchers have discovered many non-pathogenic *Geomyces* and *Pseudogymnoascus* fungi in bat hibernacula with culture-based methods [16,17]. These species complicated WNS diagnosis because their internal transcribed spacer (ITS) region sequences of the rRNA gene complex can be very similar to *P*. *destructans* [16,18]. However, more recent methods targeting the intergenic spacer (IGS) region can resolve *P*. *destructans* from its close relatives [19]. It is hypothesized that *P*. *destructans* emerged from non-pathogenic ancestors [16,20,21], so these closely related species may provide a comparative platform to study *P*. *destructans’s* adaptions for survival in hibernacula sediment. To the authors’ knowledge there are no explicit reports of culture-independent fungal community analyses in bat hibernacula, therefore the natural abundance of non-pathogenic *Pseudogymnoascus* and *Geomyces* is unknown in this environment.

Previous work has demonstrated that *P*. *destructans* can grow on complex substrates found in hibernacula [22] and at least transiently in sterilized cave sediments *in vitro* [20], but has greatly reduced saprophytic enzyme activity and growth compared to non-pathogenic *Pseudogymnoascus* [20,23]. The initial magnitude of *P*. *destructans* growth in cave sediments was positively correlated with sediment total organic carbon, but *P*. *destructans* viability decreased after 28 days in 4 out of 5 sediments tested [20]. *P*. *destructans* was recoverable from all sediments after 238 days [20], but its life cycle and stages of differentiation were not monitored. Little is known about *P*. *destructan*’s saprophytic adaptations, but a recent gene duplication of a high affinity nitrate transporter in *P*. *destructans* may contribute to its ability to survive on cave sediments [21].

The capacity to grow on substrates *in vitro* is not wholly reflective of the challenges that *P*. *destructans* must overcome as a saprophyte, as it must also compete with other microbes for space and utilizable resources. Since *P*. *destructans* is unlikely to avoid competition by quickly colonizing resources due to its slow growth [24], it may have some combination of offensive and defensive strategies to colonize saprophytic resources [25–27]. These may include the production of or resistance to diffusible antifungal molecules [27]. *P*. *destructans* is susceptible to amphotericin B, itraconazole, and ketoconazole [28], and it is likely to encounter antifungal metabolites in hibernaculum environments. Discovering how *P*. *destructans* fits into the competitive hierarchy of the heterogeneous and variable microbial communities of caves and mines [29] will help us understand if it can plausibly form self-sustaining populations in hibernaculum sediment.

Depending on the resource capture and competitive ability of *P*. *destructans*, biological control may be an effective way to reduce the conidial load of hibernaculum substrates and prevent the perpetual contamination of infected hibernacula. One strain of *Trichoderma polysporum* isolated from William Preserve Mine in New York has already been shown to outcompete *P*. *destructans* in autoclaved cave sediment, likely due to the production of diffusible antifungals [30]. Any treatment in caves or mines will be logistically challenging due to their size and inaccessibility, but biological control has an advantage over other interventions because it is potentially self-perpetuating and self-spreading. There is a risk that biological control agents will affect the microbial communities in hibernacula, but this is true of any WNS treatment. The risk of disturbing native microbial communities is mitigated because these communities in WNS-positive hibernacula have likely already shifted in response to the introduction of an invasive fungus and the loss of bats.

Theoretically, a good generalist saprophyte can utilize a diverse array of carbon sources and defend those resources from other microbes [25,27]. This study assessed the resource capture and competitive ability of *P*. *destructans* relative to non-pathogenic *Pseudogymnoascus* isolates from the Soudan Iron Mine (SM) in Tower, MN to explore the possibility that *P*. *destructans* and SM *Pseudogymnoascus* isolates share a suite of adaptations for survival in hibernaculum sediment. These experiments offer more insight into the basic biology of *P*. *destructans* and its potential to be a sustained risk in hibernaculum substrates and surfaces.

## Materials and methods

### *Pseudogymnoascus spp*. collection and propagation

All relevant protocols for bat sampling were approved by the University of Minnesota IACUC (protocol ID: 1508-32924A). Wall surface swabs, wood samples, and sediment were collected on seven levels of the Soudan Underground Mine State Park from 2013 to 2015, which was prior to the detection of WNS in Minnesota. Samples were streaked or placed onto 1.5% malt extract agar (BD Difco–Franklin Lakes, NJ) amended with 0.01 g/L of streptomycin sulfate and incubated at 10 or 22°C. *P*. *destructans* type-strain MYA-4855 was acquired from the American Tissue Type Culture Collection (ATCC), which was originally isolated from a little brown bat (*Myotis lucifugus*) in Williams Hotel Mine, NY. All *Pseudogymnoascus* spp. were maintained on Sabouraud dextrose agar (SDA), with SM *Pseudogymnoascus* isolates maintained at room temperature (~22°C) and *P*. *destructans* maintained at 10 or 15°C. Conidia were prepared for experiments by adding 2 mL of 15% glycerol to conidiated cultures, gently scraping colonies to release conidia, and then filtering the resulting conidial suspension through a sterile 5 mL pipette tip packed with cotton.

### Fungal DNA extraction, PCR, and phylogenic analysis

Phylogenic analysis was performed with the same three genes used in previous studies [19,31]. DNA was isolated from pure cultures grown on malt agar (15 g malt extract, 15 g agar, 1 L de-ionized water) using a CTAB extraction procedure described previously [32]. ITS1F/ITS4, 983F/2218R, and fRPB2-7cf/RPB2-3053br primers were used to amplify the internal transcribed spacer (ITS) region of the rRNA gene clusters, *tef1*, and *rpb2*, respectively, via PCR [33–36]. Each 25 μl PCR reaction contained ~12 ng of DNA template, 0.25 μM forward primer, 0.25 μM reverse primer, 0.05 μg/μl BSA, 1X GoTaq® green mastermix, and nuclease-free sterile water. The thermocycler programs for amplification were as follows: ITS– 94°C for 5 min, then 35 cycles of 94°C for 1 min, 50°C for 1 min, and 72°C for 1 min, and a final extension at 72°C for 5 min; *rpb2*–94 C for 5 min, then 47 cycles of 94°C for 1 min, 55.2°C for 2 min, 72°C for 2:10 min, and a final extension at 72°C for 10 min; *tef1*–94°C for 2 min, then 47 cycles of 94°C for 1 min, 55°C for 1 min, 72°C for 1:10 min, and a final extension at 72°C for 10 min. Amplicons were verified by gel electrophoresis on 1% agarose with a SYBR green 1 prestain and imaged with a Dark Reader DR45 (Clare Chemical Research–Denver, CO).

PCR products were sequenced using the same primers as above on an ABI 3730xl DNA sequencer (Applied Biosystems–Foster City, CA). Consensus sequences were assembled using Geneious 9.0 [37], and BLASTn was used to identify closely related sequences in GenBank for phylogenetic analysis. Geneious 9.0 was also used to determine phylogenetic relationships among sequences, with the MAFFT v7.222 and MrBayes 3.2.6 plugins [38,39] used for sequence alignment and Bayesian analysis, respectively. jModelTest 2.1.10 [40] was used to determine the appropriate model (JC69) for Bayesian analysis. 1.1 x 10^6^ MCMC generations were used for analysis with a sampling frequency every 200 generations, with the first 10% of sampled trees discarded as burn in. The resulting tree topologies were congruent for the analyses of each gene region, therefore sequences were concatenated and analyzed using the same methods as individual gene regions.

### *Pseudogymnoascus* colony expansion

SDA petri plates were spotted with ~2,500 *Pseudogymnoascus* spp. conidia in 10 μL of sterile water and incubated at 4°C in a walk-in cold room, at 10°C in a Danby #DBC120BLS incubator, or at room temperature in the lab (~22°C). Each *Pseudogymnoascus* sp. was spotted in quadruplicate for each temperature. Colonies were established for four days at room temperature and 10°C, and for eight days at 4°C. Thereafter, colony diameter was measured daily for 30 days by measuring length twice at right angles and taking the average.

### Substrate utilization

Filamentous fungi (FF) phenotype microarrays (Biolog–Hayward, CA; Cat# 1006) were used to compare the ability of *Pseudogymnoascus* spp. to metabolize different carbon sources per the manufacture’s protocol. These arrays are 96-well microplates that contain 95 different substrates including amines, amino acids, sugars, carboxylic acids, various polymers, and one no-substrate control. In addition to a substrate, each well supplies essential micronutrients including nitrogen, phosphorous, potassium, and sulfur. Briefly, a swab was wetted with Biolog FF-IF inoculation fluid and rolled over *Pseudogymnoascus* spp. colonies on malt extract agar to remove conidia and mycelium fragments. Swab contents were diluted to 0.14 OD_600_ in inoculation fluid and 200 μL were transferred to each well in triplicate FF arrays. Arrays were incubated and shaken at 15°C.

Growth was evaluated at OD_750_ using an EL800 microplate reader (Bio-Tek–Winooski, VT) after seven days for SM *Pseudogymnoascus* isolates and after 14 days for *P*. *destructans*. Values were restricted to 0.0–2.0 OD_750_ in all analyses to account for the linear range of the microplate reader. It was empirically determined that 7 and 14 days would result in a reasonable comparison since SM *Pseudogymnoascus* isolates grow approximately twice as fast as *P*. *destructans* at 10°C.

Niche width was defined as the number of substrates utilized in the FF arrays, while growth efficiency was defined as the mean OD_750_ of wells with utilized substrates [41]. Since some growth occurred in the no-substrate control wells, pairwise one-tailed Welch’s t-tests were used to determine a utilization threshold on a substrate-by-substrate basis instead of an absolute OD_750_ cutoff applied to all substrates. A one-tailed test was appropriate because growth promotion, not growth inhibition, is relevant to establishing a utilization threshold and maximizes statistical power for a given alpha. A substrate was considered “utilized” if the mean growth on a substrate was statistically greater than mean control growth (p < 0.05). Pair-wise, two tailed Welch’s t-tests were used to compare growth efficiency (mean growth) among *Pseudogymnoascus* spp. at the 95% confidence level.

### Susceptibility testing

Disk-diffusion assays were performed on 50 mL SDA in 150 x 15 mm petri plates. Sterile filter paper disks were prepared with 6, 3, 1.5, 0.375, and 0.18 μg of amphotericin B (MP Biomedicals–Santa Ana, CA), caspofungin, itraconazole, or ketoconazole (LTK Laboratories–St. Paul, MN) in methanol. These antifungals were selected to represent different classes of systemic antifungals found in nature (polyenes, echinocandins, and azoles). Duplicate plates were spread-inoculated with ~4,000 *Pseudogymnoascus* spp. conidia in 200 μL of sterile water and allowed to dry. Antifungal disks were placed on dry plates and plates were incubated at 4 or 15°C. Minimum inhibitory concentrations (MIC) of each antifungal were determined visually after two and three weeks for SM *Pseudogymnoascus* isolates and *P*. *destructans* at 15°C, respectively, and after three and five weeks for SM *Pseudogymnoascus* isolates and *P*. *destructans* at 4°C, respectively.

### Competition assays between SM *Pseudogymnoascus* isolates and *P*. *destructans*

Agar plugs (5 mm^3^) were cut from the edge of established *Pseudogymnoascus* spp. colonies and placed ~1 cm apart on SDA and incubated at 10°C for four weeks in duplicate. Inhibition of *P*. *destructans* growth was qualitatively evaluated by visually assessing the size of control and competing *P*. *destructans* colonies.

### HPLC analysis and anti-*P*. *destructans* activity of SM *Pseudogymnoascus* extracts

SM *Pseudogymnoascus* isolates were grown on rice (10g of rice autoclaved in 12 mL water) at 10°C for 30 days. Cultures were extracted overnight with two rounds of 100 mL methanol followed by two rounds of 100 mL ethyl acetate. Extracts from the same culture were combined and dried under reduced pressure. Supelco 3 mL DSC-18 (C_18_) solid-phase extraction columns (Sigma-Aldrich–St. Louis, MO) were used to clean up samples prior to disk diffusion and HPLC analysis. Extract residues were re-dissolved in 6 mL 5% methanol and bound to pre-equilibrated columns, washed with two volumes of 5% methanol, and eluted with two volumes of 100% methanol. Eluted metabolites were dried and re-suspended to 1 mg/mL in methanol for reversed-phase HPLC analysis on an Agilent 1200 system with an Eclipse XDB-C18 column (5 μm, 4.6 x 150 mm). Metabolites were eluted at 1 mL/min with 10% acetonitrile for 1.5 mins, followed by a linear gradient from 10% to 95% acetonitrile over 20 mins, a hold at 95% acetonitrile for 2 mins, a linear gradient from 95% to 100% acetonitrile over 3 mins, and finally re-equilibration to 10% acetonitrile over 2 mins. Chromatographic peaks were detected by diode array from 200 to 600 nm in 10 nm increments.

Disk-diffusion assays were performed on 15 mL SDA in 100 x 10 mm petri plates to assess the anti-*P*. *destructans* activity of SM *Pseudogymnoascus* rice culture extracts. Filter paper disks were prepared with 0.5 mg SPE-cleaned extract, placed on plates spread inoculated with ~2,500 *P*. *destructans* conidia, and incubated at 10°C for three weeks. Assays were performed in triplicate.

## Results

### SM *Pseudogymnoascus* are closely related to other *Pseudogymnoascus* spp. isolated from hibernacula

**Fig 1:**
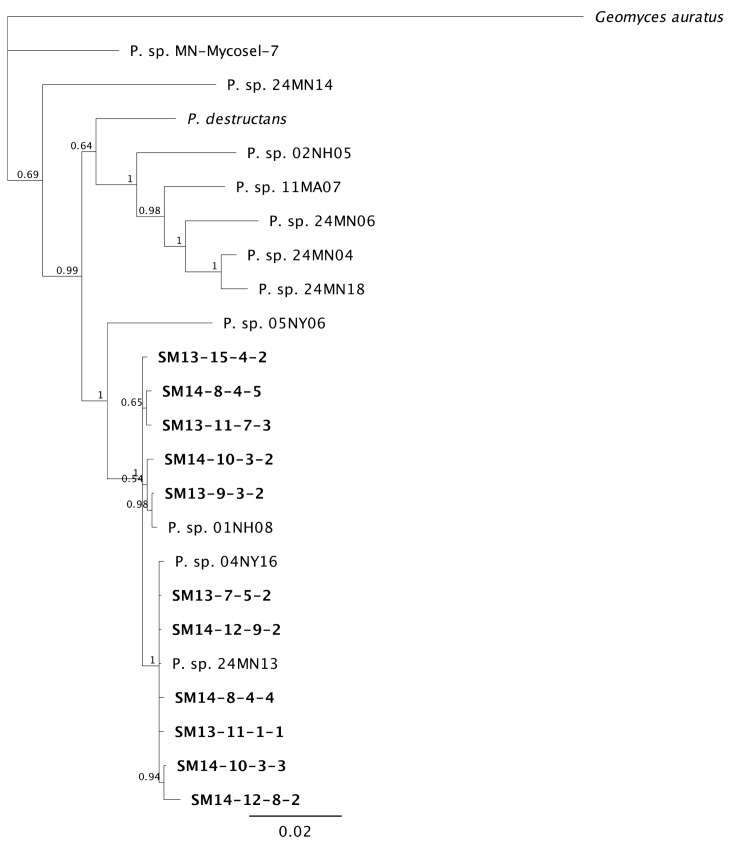
Phylogenetic tree of *Pseudogymnoascus* isolates obtained from the Soudan Iron Mine (SM) in Minnesota. Tree was constructed using Bayesian analysis of combined ITS, EF-1 and RPB2 gene regions from *Pseudogymnoascus* species. Soudan Mine (SM) isolates are indicated in bold. SM isolates group with Genbank *Pseudogymnoascus* sequences reported by other investigations in New Hampshire, New York, and Minnesota. Posterior probabilities >50 are shown at branch nodes. *Geomyces auratus* served as the outgroup.

A three-gene phylogeny of *Pseudogymnoascus* species shows close similarity among isolates from the different mine shaft levels (Fig 1). SM isolates group with the previously described *P*. *verrucosus* complex, matching isolates from NH, NY and MN hibernacula [31]. Overall, the SM isolates separated into two clades with SM13-7-5-2, SM14-12-9-2, SM14-8-4-4, SM13-11-1-1, SM14-10-3-3, SM14-12-8-2 separate from SM13-15-4-2, SM14-8-4-5, SM13-11-7-3, SM14-10-3-2, SM13-9-3-2. However, short branch lengths indicate minor base changes among these isolates. All gene sequences used for phylogenic analysis have been deposited in Genbank (S1 Table).

### SM *Pseudogymnoascus* colonies expand faster and at higher temperature than *P*. *destructans* colonies

**Fig 2:**
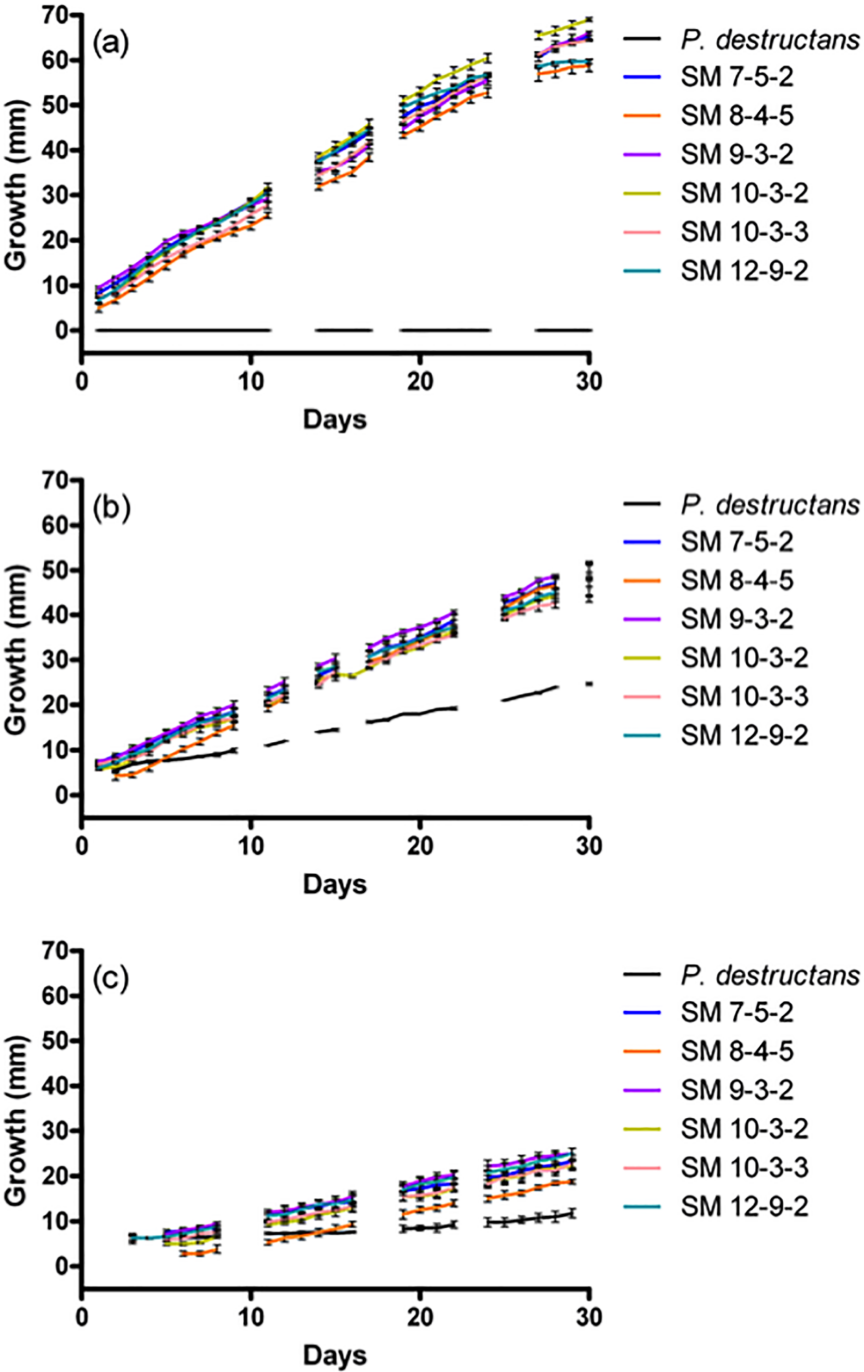
Colony expansion of *Pseudogymnoascus* spp. on SDA over 30 days. Diameter of *Pseudogymnoascus* spp. colonies at various temperatures over time (a) 22°C, (b) 10°C, (c) 4°C. Error bars represent standard error of averaged replicates.

Expansion rates were similar among SM *Pseudogymnoascus* isolates at all temperatures, and these organisms are subsequently referred to as one group (Fig 2A–2C). SM *Pseudogymnoascus* colonies reached diameters of ~60 mm after 30 days at room temperature, with no *P*. *destructans* growth as expected [24] (Fig 2A). SM *Pseudogymnoascus* colonies reached diameters of ~50 mm at 10°C after 30 days on SDA, while *P*. *destructans* colonies achieved a diameter of 25 mm (Fig 2B). Differences in growth were minimized at 4°C, with SM *Pseudogymnoascus* colonies reaching diameters ~25 mm and *P*. *destructans* colonies reaching diameters of 13 mm (Fig 2C).

### SM *Pseudogymnoascus* isolates utilized more substrates more quickly than *P*. *destructans*

Biolog filamentous fungi (FF) phenotype microarrays were inoculated with SM *Pseudogymnoascus* isolates or *P*. *destructans* to compare their substrate utilization capacities. Each well in the plate contains a single carbon source with essential micronutrients, and the capacity to utilize different substrates can be determined by measuring fungal growth in each well. The utilization of different sugars, amino acids, carboxylic acids, and polymers can be summarized by ‘niche width’ (the total number of utilized substrates, ranging 0–95) and ‘growth efficiency’ (mean growth in wells with utilized substrates) [41]. The full Biolog FF array dataset can be found in S1 Text.

**Fig 3:**
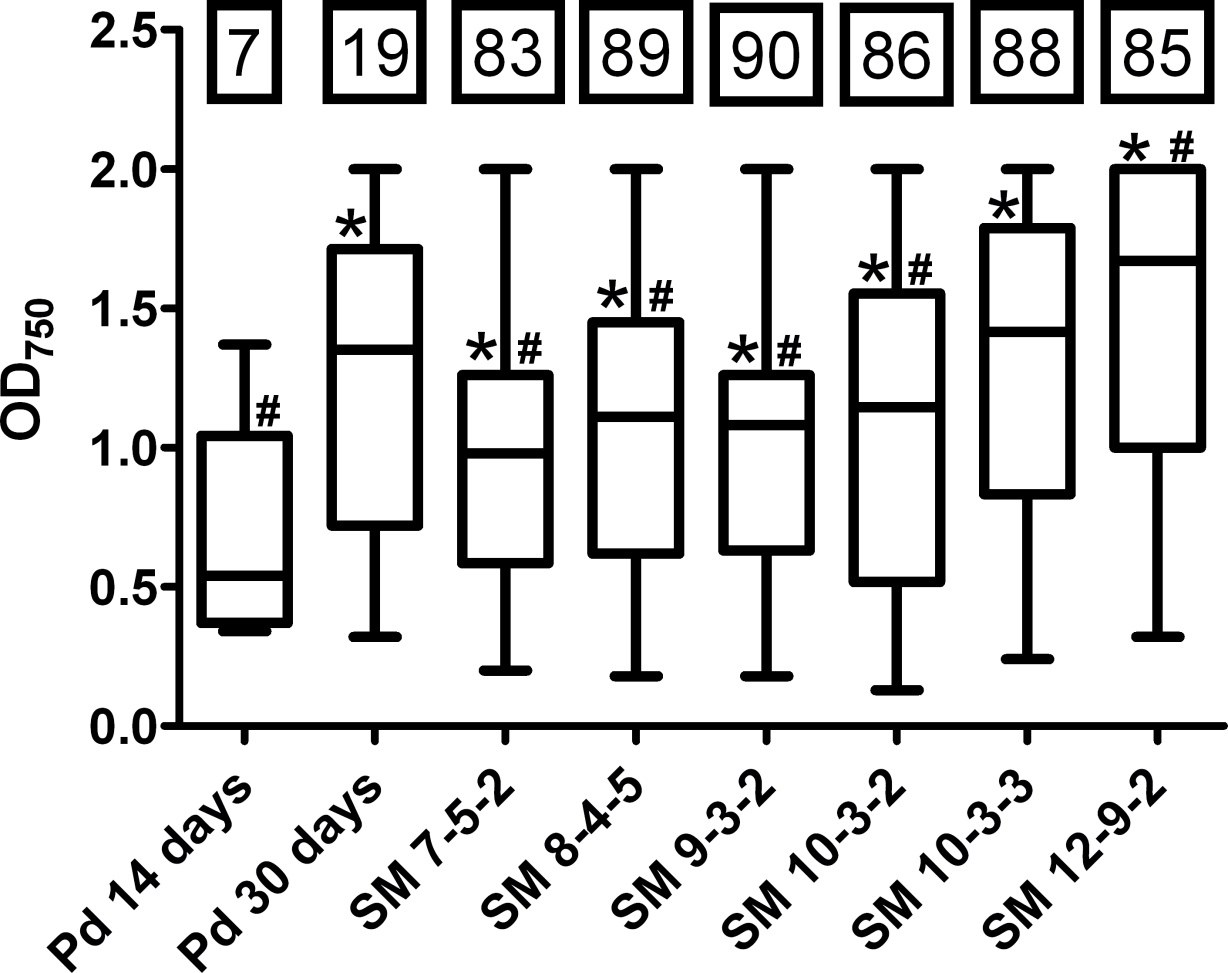
Niche width and growth efficiency of *Pseudogymnoascus* spp. at 15°C. The number of substrates utilized by each strain (niche width) in Biolog FF phenotype mircoarrays is indicated in black boxes. OD_750_ of wells with utilized substrates is displayed, where the mean OD_750_ is each strain’s growth efficiency. SM *Pseudogymnoascus* isolates were evaluated after 7 days of growth, while *P*. *destructans* was evaluated after 14 and 30 days of growth. Statistical differences (p < 0.05) are indicated by * and # for pair-wise Welch’s t-tests against 14 day and 30 day-*P*. *destructans*, respectively. Pd = *P*. *destructans*.

Niche widths were ~87 for all SM *Pseudogymnoascus* isolates after 7 days of growth, compared to a niche width of 7 for *P*. *destructans* after 14 days (Fig 3). SM *Pseudogymnoascus* isolates grow approximately twice as fast as *P*. *destructans* at 10°C (Fig 2), therefore 7 and 14 days were chosen as comparable endpoints. The niche width of *P*. *destructans* increased to 19 after 30 days of growth; however, this was still well below the niche widths achieved by the SM *Pseudogymnoascus* isolates after only 7 days.

Growth efficiencies of all SM *Pseudogymnoascus* isolates were significantly higher than the efficiency of 14 day-*P*. *destructans* (p < 0.05) (Fig 3, S2 Table). The growth efficiency of *P*. *destructans* greatly increased over time, with the efficiency of 30 day-*P*. *destructans* matching SM 10-3-3 and significantly exceeded that of SM 7-5-2, SM 8-4-5, SM 9-3-2, and SM 10-3-2 (p < 0.05) (Fig 3, S2 Table). The growth efficiency of 30-day *P*. *destructans* was still lower than that of SM 12-9-2 (p < 0.05) (Fig 3, S2 Table).

**Fig 4:**
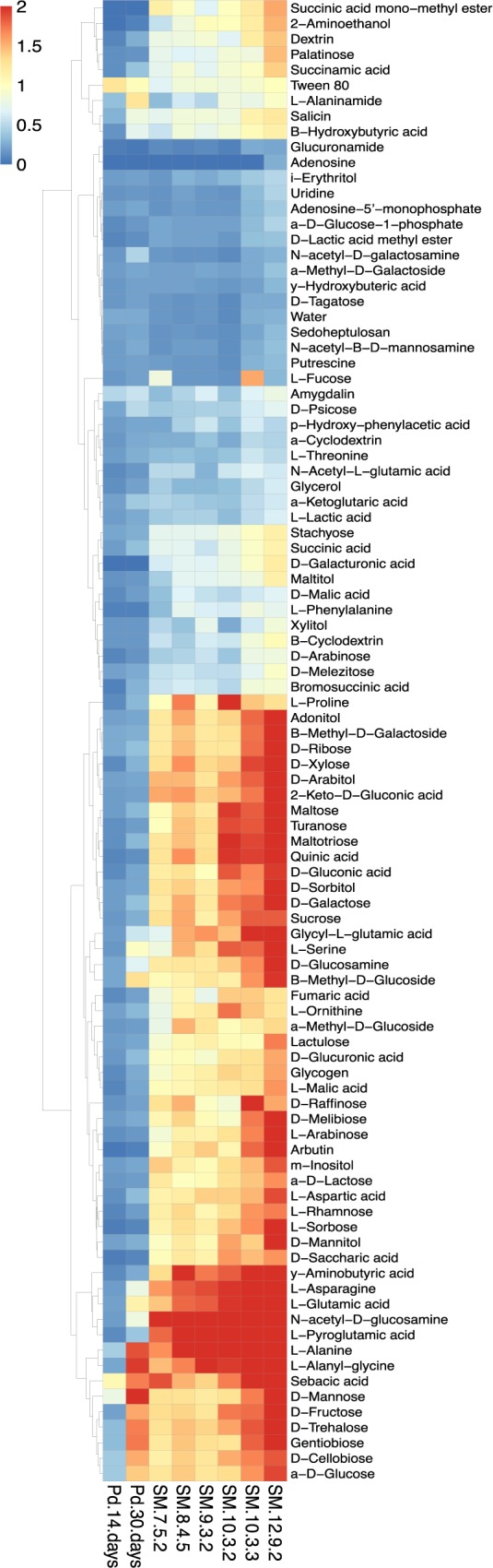
Heatmap of *Pseudogymnoascus* spp. substrate utilization at 15°C. Rows correspond to average growth (OD_750_) in wells of Biolog FF phenotype microarrays, while columns correspond to different *Pseudogymnoascus* spp. SM *Pseudogymnoascus* isolates were evaluated after 7 days of growth, while *P*. *destructans* was evaluated after 14 and 30 days of growth. Rows are clustered by k-means to display patterns of utilization.

Fig 4 displays the utilization of specific substrates in the FF arrays by *Pseudogymnoascus* spp. SM *Pseudogymnoascus* isolates utilized a wide, but very similar, variety of sugars, carboxylic acids, amino acids, and other substrates with no clear metabolic preferences (Fig 4). The notable exception to their similar utilization profiles was L-fucose, which was only utilized by SM 7-5-2 and 10-3-3 (Fig 4). Growth efficiencies on particular substrates among the SM *Pseudogymnoascus* isolates differed greatly, with SM 12-9-2 typically being the most efficient isolate (Fig 4).

*P*. *destructans* utilized only simple sugars, lipid-like compounds, and amino acids after 14 days (Fig 4). *P*. *destructans* expanded its utilization of simple sugars and amino acids after 30 days, but also started to utilize small carboxylic acids and amino sugars (Fig 4). Even though *P*. *destructans* had a much smaller niche width than the SM *Pseudogymnoascus* isolates (Fig 3), it was the exclusive utilizer of N-acetyl-D-galactosamine (Fig 4). The lipid-like compounds (Tween 80, sebacic acid, and amygdalin) were the only substrates where *P*. *destructans*’s growth efficiency matched or exceeded those of the SM *Pseudogymnoascus* isolates after 14 days (Fig 4).

### *P*. *destructans* is more susceptible to antifungals than SM *Pseudogymnoascus* isolates, but susceptibility varies with temperature

*P*. *destructans* was susceptible to amphotericin B, itraconazole, and ketoconazole (Table 1) [28]. While its susceptibility to itraconazole and ketoconazole did not change (MIC < 0.18 and MIC = 1.5 μg, respectively), its susceptibility to amphotericin B increased at higher temperature (MIC = 1.5 μg at 4°C vs. MIC < 0.18 μg at 15°C) (Table 1). *P*. *destructans* was resistant to caspofungin within our concentration range (MIC > 6 μg) as previously reported [28], and there was no noticeable change in susceptibility with temperature.

**Table 1:** Antifungal susceptibility of *Pseudogymnoascus* spp.

	*Pseudogymnoascus* spp. MIC (μg)													
	*P*. *destructans*		SM 7-5-2		SM 8-4-5		SM 9-3-2		SM 10-3-2		SM 10-3-3		SM 12-9-2	
Antifungal	4°C	15°C	4°C	15°C	4°C	15°C	4°C	15°C	4°C	15°C	4°C	15°C	4°C	15°C
Caspofungin	> 6	> 6	3	> 6	3	> 6	6	> 6	3	> 6	3	> 6	1.5	> 6
Amphotericin B	1.5	< 0.18	> 6	> 6	> 6	> 6	> 6	> 6	> 6	> 6	> 6	> 6	> 6	> 6
Itraconazole	< 0.18	< 0.18	1.5	1.5	1.5	3	1.5	1.5	0.375	3	0.375	3	1.5	1.5
Ketoconazole	1.5	1.5	> 6	6	> 6	6	> 6	3	6	6	> 6	> 6	> 6	3

MICs were determined as the lowest concentration of antifungal that did not produce a visible zone of inhibition in disk-diffusion assays. Plates incubated at 4°C were evaluated after three weeks for SM *Pseudogymnoascus* isolates and after five weeks for *P*. *destructans*. Plates incubated at 15°C were evaluated after two weeks for SM *Pseudogymnoascus* isolates and after three weeks for *P*. *destructans*.

SM *Pseudogymnoascus* isolates were more resistant to amphotericin B, itraconazole, and ketoconazole relative to *P*. *destructans* at 4 and 15°C (Table 1). Unlike *P*. *destructans*, SM *Pseudogymnoascus* susceptibility to amphotericin B did not noticeably change with temperature *Pseudogymnoascus*(Table 1). SM *Pseudogymnoascus* isolates were most susceptible to itraconazole, but their MICs were at least an order of magnitude higher than those observed for *P*. *destructans* (Table 1). SM isolates 10-3-2 and 10-3-3 were exceptions to this trend with MIC = 0.375 μg against itraconazole (Table 1). Azole susceptibility differentially varied with temperature for some SM *Pseudogymnoascus* isolates, with itraconazole and ketoconazole showing opposite trends (Table 1). SM isolates 8-4-5, 10-3-2, and 10-3-3 were less susceptible to itraconazole at higher temperature (MIC = 1.5 or 0.375 μg at 4°C vs. MIC = 3 μg at 15°C), but itraconazole susceptibility did not change for SM 7-5-2, 9-3-2, or 12-9-2 (MIC = 1.5 μg) (Table 1). SM isolates 9-3-2 and 12-9-2 were more susceptible to ketoconazole at higher temperature (MIC > 6 μg at 4°C vs. MIC = 3 μg at 15°C), with SM 7-5-2 and 8-4-5 showing potentially smaller increases in ketoconazole susceptibility (MIC > 6 μg at 4°C vs. MIC = 6 μg at 15°C) (Table 1). Ketoconazole susceptibility did not noticeably change for SM 10-3-2 or 10-3-3 (MIC = 6 and > 6 μg, respectively) (Table 1). All SM *Pseudogymnoascus* isolates were more susceptible to caspofungin at lower temperatures (Table 1).

### SM *Pseudogymnoascus* outcompetes *P*. *destructans* in co-culture

**Fig 5:**
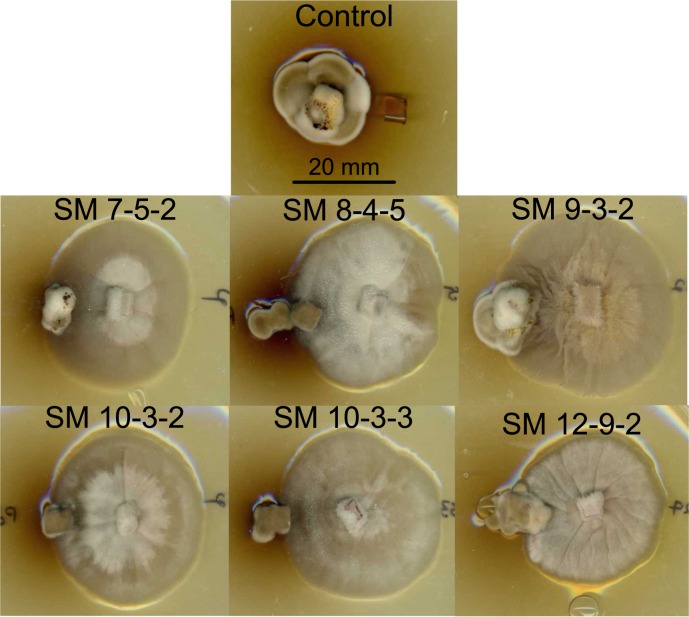
Competition of SM *Pseudogymnoascus* and *P*. *destructans* colonies. Plug competition assay after four weeks of growth on SDA at 15°C. *P*. *destructans* colonies are on the left of each panel, while SM *Pseudogymnoascus* colonies are on the right. The control was a *P*. *destructans* colony grown in the presence of an uninoculated agar block.

All SM *Pseudogymnoascus* colonies limited the expansion of *P*. *destructans* colonies after four weeks of incubation at 10°C (Fig 5). *P*. *destructans* colonies expanded more against SM 8-4-5, 9-3-2, and 12-9-2 colonies than against 7-5-2, 10-3-2, and 10-3-3 colonies (Fig 5).

### SM *Pseudogymnoascus* isolates produce more extractable metabolites in culture, with some having anti-*P*. *destructans* activity

**Fig 6:**
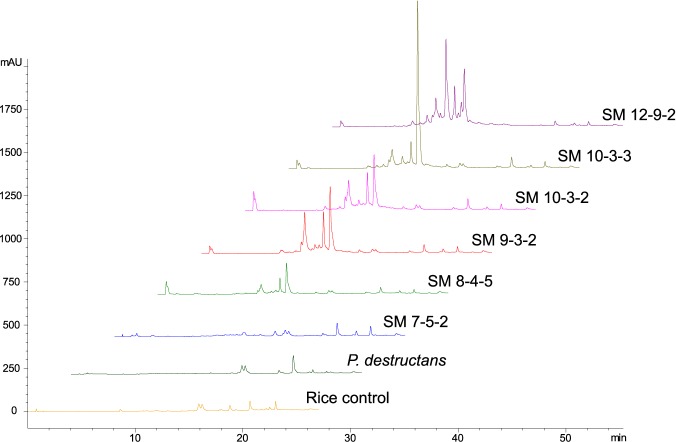
Reversed-phase HPLC chromatograms of *Pseudogymnoascus* spp. rice culture extracts. All extracts were normalized to 1 mg/mL, and chromatographic peaks at 220 nm are displayed. The control was an extract of uninoculated rice.

Most SM *Pseudogymnoascus* isolates had a similar diversity of extractable semi-polar metabolites when grown on rice at 10°C (Fig 6, S2 Fig). SM isolate 7-5-2 and *P*. *destructans* did not appear to produce any extractable metabolites under the experimental conditions (Fig 6, S2 Fig). Three SM *Pseudogymnoascus* extracts showed anti-*P*. *destructans* activity in disk diffusion assays (Table 2). Extracts from SM isolates 10-3-2 and 10-3-3 produced small zones that were sustained over three weeks, while the 12-9-2 extract produced a large initial zone that receded by week three (Table 2). Pictures of disk-diffusion assays can be found in S1 Fig.

**Table 2:** Anti-*P*. *destructans* activity of SM *Pseudogymnoascus* extracts.

Extract	SM 7-5-2	SM 8-4-5	SM 9-3-2	SM 10-3-2	SM 10-3-3	SM 12-9-2
Zone size (2 wk)	-	-	-	+	+	+++
Zone size (3 wk)	-	-	-	Minimal	Minimal	+

Disk diffusion assays were performed in triplicate to test the activity of SM *Pseudogymnoascus* extracts against *P*. *destructans*. Inhibitory activity was quantified as a zone of inhibition, with (+) indicating zones 3–20 mm in diameter and (-) indicating no inhibition. Disks contained 0.5 mg of rice culture extracts from SM isolates. Plates were incubated at 15°C and evaluated after two and three weeks. Pictures of disk diffusion assays can be found in S1 Fig.

## Discussion

White-nose syndrome has spurred research on the microbiome of bat hibernacula to better understand the biology of *P*. *destructans*. Many *Pseudogymnoascus* and *Geomyces* species have been discovered in WNS-positive hibernacula [16,31], but our *Pseudogymnoascus* isolates represent the SM fungal community prior to the invasion of *P*. *destructans*. SM isolates separated into two distinct clades and grouped with other *Pseudogymnoascus* from the *P*. *verrucosus* complex in a three-gene phylogeny, although short branch lengths indicated only minor differences among the isolates (Fig 1). We collected physiological data from a subset of each clade, but there was no clear association between clades and our physiological data. *P*. *destructans* was both genetically and physiologically distinct from all SM *Pseudogymnoascus* isolates.

The first important distinction between the SM *Pseudogymnoascus* isolates and *P*. *destructans* is their ability to grow at room temperature (Fig 2). While *P*. *destructans* is psychrophilic with a growth optimum of ~15°C and upper critical temperature of ~19°C [24], the SM *Pseudogymnoascus* isolates were psychrotolerant with the ability to grow at both room and low temperature (Fig 2). Even though all SM *Pseudogymnoacus* isolates grew best at room temperature (~22°C), they still outgrew *P*. *destructans* at 4 and 10°C (Fig 2). For reference, temperatures where most bats can be found in the Soudan Mine (level 12 and 27) are generally 2–10°C during the hibernation period. Even though their growth rates were clearly different, neither *P*. *destructans* nor SM isolates displayed expansion that could be characterized as “fast”. In general, slow growth may be an important adaptation in oligotrophic environments, such as caves [42].

*P*. *destructans* is an opportunistic or facultative pathogen of hibernating bats that takes advantage of its host’s decreased immune function during torpor [43]. Since torpor is a reliably reoccurring state, *P*. *destructans* may have metabolic requirements more like an obligate pathogen because immunosuppression due to torpor is not an abnormal or rare host state. If *P*. *destructans* is a facultative pathogen in the environment, it may have resource capture traits similar to non-pathogenic *Pseudogymnoascus* spp. The results presented here show *P*. *destructans* had a reduced niche width compared to SM *Pseudogymnoascus* isolates in substrate utilization assays (Fig 3), suggesting it is more specialized than its non-pathogenic relatives. This conclusion is further supported by previous reports that *P*. *destructans* has reduced saprophytic enzyme activity compared to non-pathogenic *Pseudogymnoascus* spp. [23]. Since the nutrients available to microbes in different caves and mines are highly variable and heterogeneously distributed in space and time [29], it is difficult to generalize which nutrients are ecologically relevant to saprophytic survival. The Biolog substrates provide a general view of what substrates may be utilized, but additional study is needed to identify compounds/nutrients being utilized in specific caves and mines.

The overall conversion of substrate to biomass should be maximized at 15°C for *P*. *destructans* [24], but not for the SM *Pseudogymnoascus* isolates (Fig 2). Lipid-like compounds (Tween 80, sebacic acid, and amygdalin) were the only substrates used by *P*. *destructans* after 14 days with efficiencies comparable to those of the SM *Pseudogymnoascus* isolates (Fig 4), which supports its nutritional preference for lipids [22]. *P*. *destructans* drastically increased its growth efficiency over time (Figs 3 and 4) and utilized its limited panel of substrates as effectively as the SM *Pseudogymnoascus* isolates after 30 days. Even though the SM *Pseudogymnoascus* isolates were genetically distinct (Fig 1), they had very similar utilization profiles and growth efficiencies (Figs 3 and 4). This is good evidence these isolates are occupying similar nutritional niches within the mine. Overall, *P*. *destructans* is unlikely to occupy the same generalist saprophyte role presumably occupied by non-pathogenic *Pseudogymnoascus* spp. due to its compressed niche width and much slower conversion of substrates to biomass.

*P*. *destructans* can grow and reproduce on some complex substrates *in vitro* [22], but it certainly faces competition for these resources in hibernacula sediment. Attack and defense strategies are important to displace other microbes from contested resources or to avoid displacement, respectively [27]. Since *P*. *destructans* grows very slowly relative to other fungi [24] (Fig 2A–2C), it is unlikely to avoid attack by rapidly colonizing resources and may require robust defense strategies in hibernaculum substrates. The plasma membrane is a major antifungal target that changes composition in response to temperature, therefore environmental temperatures are important considerations in microbial defense. Using *Geomyces pannorum* as a comparative model in the literature, we expected increased susceptibility to polyenes and azoles at higher temperature when ergosterol is more abundant in the plasma membrane [44]. However, this trend was only apparent for amphotericin B vs. *P*. *destructans* and ketoconazole vs. some SM *Pseudogymnoascus* isolates (Table 1). The efficiency of resistance mechanisms may also vary with temperature, which could explain the decrease in itraconazole susceptibility observed for some SM *Pseudogymnascus* isolates at higher temperature and the lack of azole response in *P*. *destructans*. To the authors’ knowledge, changes in the cell wall are not a known adaptation of psychrophilic or psychrotolerant fungi [45,46], so we did not expect the observed change in susceptibility to caspofungin (Table 1). Just considering these variations in antifungal susceptibility, *P*. *destructans* may be more easily displaced by polyene and azole-producing microbes compared to the SM *Pseudogymnoascus* isolates, but is better defended against echinocandin-producing organisms at low temperature. Future work could focus on the interplay between temperature, membrane/cell wall structure, and resistance mechanisms of *Pseudogymnoascus* spp.

*P*. *destructans* and other *Pseudogymnoascus* spp. may encounter each other in hibernaculum substrates and compete for space, if not resources. All SM *Pseudogymnoascus* isolates outcompeted *P*. *destructans* on nutrient-rich media (Fig 5), but this was not surprising based on their faster growth and superior resource capture ability. A slower growing, more specialized organism could use attack strategies to outcompete faster growing generalists, but this was not true for *P*. *destructans* under our conditions. Assays on artificial media are not always reflective of competition in the environment [27], so field studies are needed to confirm this behavior. It is important to consider how interactions among *P*. *destructans* and non-pathogenic *Pseudogymnoascus* spp. occur in an oligotrophic subterranean environment.

Competition assays could not be used to determine if growth inhibition was a result of direct antagonism or indirect nutrient limitation, so each SM *Pseudogymnoascus* isolate was extracted to assess the production of anti-*P*. *destructans* metabolites. All SM *Pseudogymnoascus* extracts (except 7-5-2) produced similar HPLC profiles (Fig 6), but only extracts of SM 10-3-2, 10-3-3, and 12-9-2 inhibited *P*. *destructans* growth (Table 2) demonstrating these isolates have some offensive mechanisms effective against *P*. *destructans*. Interestingly, the activity of these extracts was transient (Table 2), indicating low stability of active metabolites or delayed defensive mechanisms by *P*. *destructans*.

*P*. *destructans* is likely susceptible to biological control agents in hibernacula sediments considering its demonstrated growth (Fig 2), resource capture (Figs 3 and 4), and competitive ability (Tables 1 and 2, Fig 5). Several *Trichoderma* isolates from cave sediments capable of preventing *P*. *destructans* conidia from germinating in cave sediments have been identified [30], and non-pathogenic *Pseudogymnoascus* spp. may be another common community member that can help control *P*. *destructans*. A multi-species pool of biological control candidates is useful because it is unlikely a single organism will provide a good solution for all hibernacula due to differing mineral compositions, nutrient availability, and moisture levels. However, the WNS community needs to better understand the risk of *P*. *destructans* infection from environmental reservoirs to correctly implement any biological control scheme. It is important to consider that *P*. *destructans* and non-pathogenic *Pseudogymnoascus* spp. are part of larger microbial communities with complex interactions that may facilitate or prevent the establishment of *P*. *destructans*. This work focused on non-pathogenic *Pseudogymnoascus* spp. because they may reveal traits important for *P*. *destructans* survival in hibernaculum substrates, but the relative importance of their interactions within the microbial community is unknown.

We initially hypothesized that SM *Pseudogymnoascus* isolates could be used as a proxy for *P*. *destructans* in antifungal assays due to their close genetic relationship and ability to grow rapidly at room temperature. However, our phenotypic studies revealed that *P*. *destructans* is remarkably different from SM *Pseudogymnoascus* isolates. These phenotypic differences are likely reflections of traits that help non-pathogenic *Pseudogymnoascus* spp. thrive as generalist saprophytes and traits that allow *P*. *destructans* to thrive as a bat pathogen. While *P*. *destructans* may have the capacity to obtain some saprophytically-derived nutrients [22,23] (Figs 3 and 4), it was more specialized and less competitive than SM *Pseudogymnoascus* isolates (Tables 1 and 2, Fig 5). This does not mean that *P*. *destructans* cannot or does not form stable populations in hibernacula sediment and surfaces, but that it must do so differently than its non-pathogenic relatives.

## Supporting information

S1 Fig Pictures of disk diffusion plates summarized in Table 2. Disks contained 0.5 mg of HPLC-ready extract and plates were incubated at 15°C and evaluated after (a) two and (b) three weeks.
(TIFF)

S2 Fig Reversed-phase HPLC of *Pseudogymnoascus* rice culture extracts. Chromatographic peaks were detected by diode array. The control was an extract of uninoculated rice, and all extracts were normalized to 1 mg/mL. All SM *Pseudogymnoascus* produced more detectible semi-polar metabolites in rice culture compared to *P*. *destructans*, with the exception of SM 7-5-2.
(PDF)

S1 Table GenBank numbers of *Pseudogymnoascus* isolates used in antagonism studies and phylogenetic analysis (in bold) and those obtained from GenBank for phylogenetic comparison. (PDF)

S2 Table Pair-wise Welsh’s t-test results for comparisons of growth efficiency. See the materials and methods for t-test details, values are rounded to the nearest non-zero integer.
(PDF)

S1 Text Biolog plate guide and data. (XLSX)
