# Supplementary figures and images for: Resource capture and competitive ability of non-pathogenic Pseudogymnoascus spp. and P. destructans, the cause of white-nose syndrome in bats
Source: PLoS One. 2017 Jun 15;12(6):e0178968. doi: 10.1371/journal.pone.0178968 (PMC5472292; doi:10.1371/journal.pone.0178968)

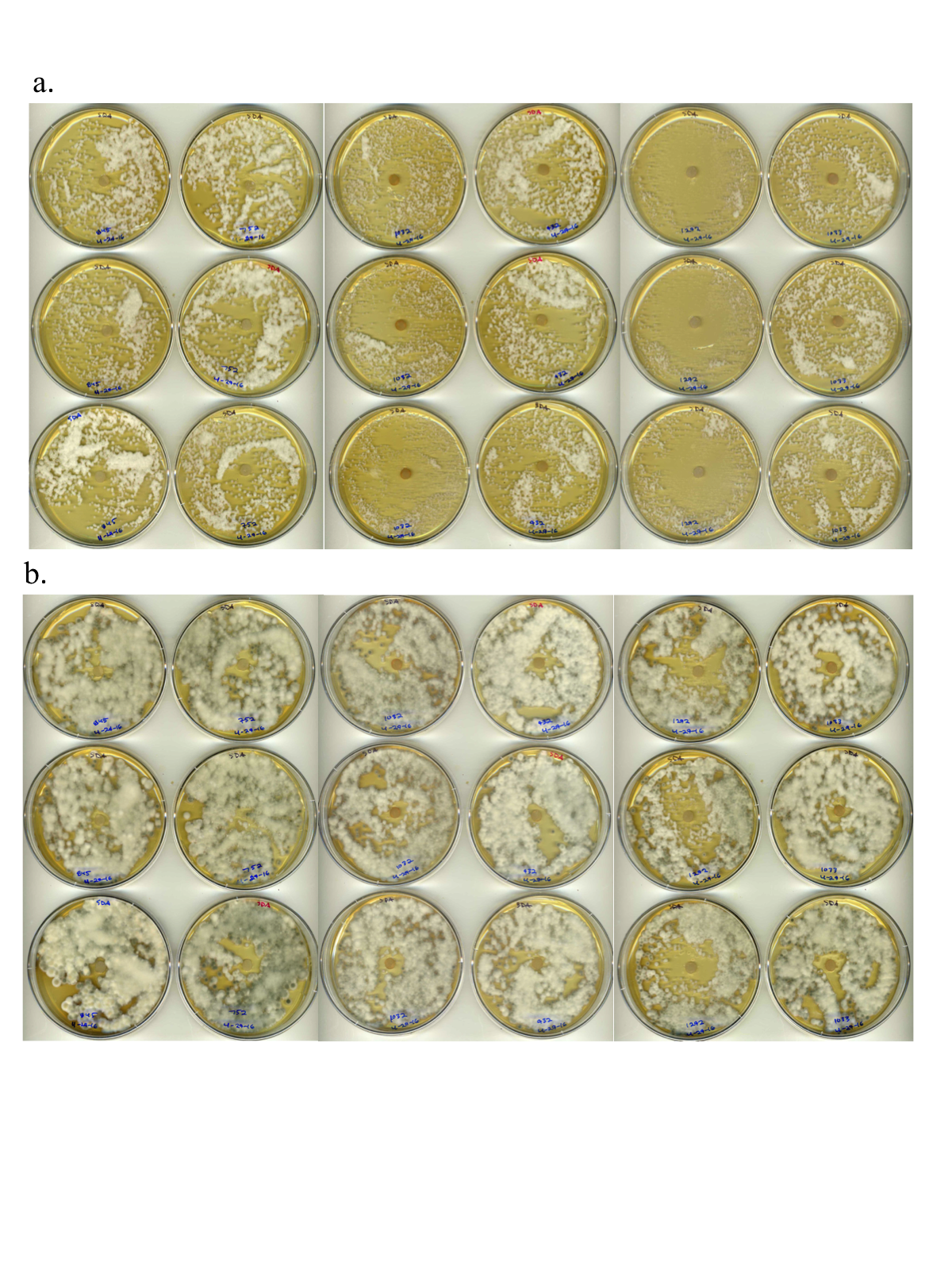

Supplement: S1 Fig — Disks contained 0.5 mg of HPLC-ready extract and plates were incubated at 15°C and evaluated after (a) two and (b) three weeks. (TIFF) [file pone.0178968.s001.tiff]

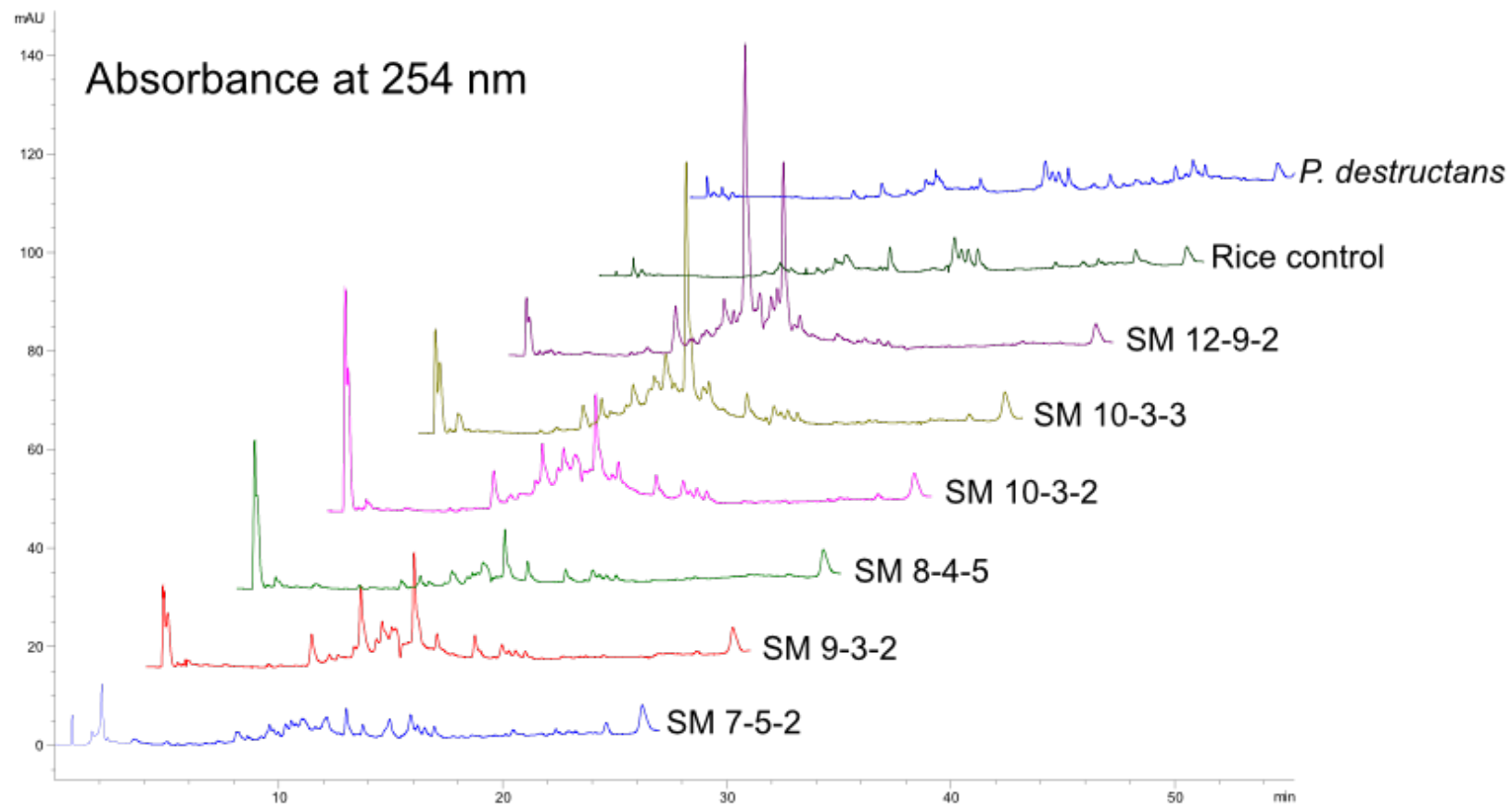

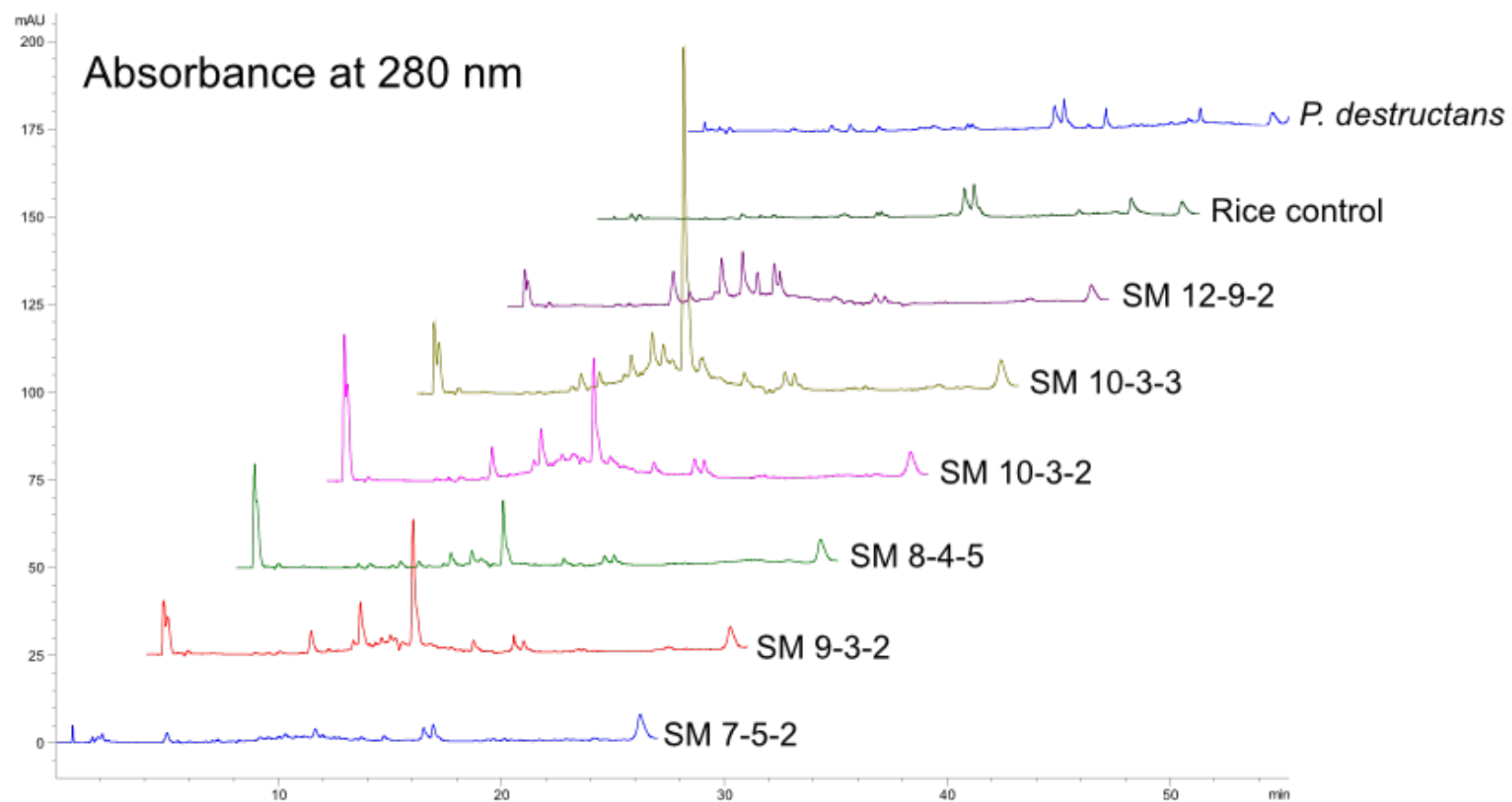

Supplement: S2 Fig — Chromatographic peaks were detected by diode array. The control was an extract of uninoculated rice, and all extracts were normalized to 1 mg/mL. All SM Pseudogymnoascus produced more detectible semi-polar metabolites in rice culture compared to P. destructans, with the exception of SM 7-5-2. (PDF) [file pone.0178968.s002.pdf]
