# Supplementary material for: Resource capture and competitive ability of non-pathogenic Pseudogymnoascus spp. and P. destructans, the cause of white-nose syndrome in bats
Source: PLoS One. 2017 Jun 15;12(6):e0178968. doi: 10.1371/journal.pone.0178968 (PMC5472292; doi:10.1371/journal.pone.0178968)
Supplement: S1 Table — (PDF) [file pone.0178968.s003.pdf]

	**GenBank #**		
**Isolate**	**ITS region**	**RPB2**	**TEF1**
**SM13-7-5-2**	KY270892	KY270903	KY270881
**SM13-9-3-2**	KY270893	KY270904	KY270882
**SM13-11-1-1**	KY270894	KY270905	KY270883
**SM13-11-7-3**	KY270895	KY270906	KY270884
**SM13-15-4-2**	KY270897	KY270907	KY270885
**SM14-8-4-4**	KY270898	KY270908	KY270886
**SM14-8-4-5**	KY270899	KY270909	KY270887
**SM14-10-3-2**	KY270900	KY270910	KY270888
**SM14-10-3-3**	KY270901	KY270911	KY270889
**SM14-12-8-2**	KY270896	KY270912	KY270890
**SM14-12-9-2**	KY270902	KY270913	KY270891
P. sp. 24MN13	JX270621	KF017743	KF017802
P. sp. 04NY16	JX270377	KF017707	KF017762
P. sp. 01NH08	JX270343	KF017701	KF017756
	**GenBank #**		
**Isolate**	**ITS region**	**RPB2**	**TEF1**
P. sp. 05NY06	JX270385	KF017709	KF017764
P. sp. 24MN18	JX270626	KF017745	KF017804
P. sp. 24MN04	JX270612	KF017741	KF017800
P. sp. 24MN06	JX270614	KF017742	KF017801
P. sp. 11MA07	JX270442	KF017719	KF017774
P. sp. 02NH05	JX270350	KF017703	KF017758
P. sp. 24MN14	JX270622	KF017744	KF017803
P. spMN-Mycosel-7	KF039899	KF017753	KF017813
P. destructans	EU884921	KF017747	KF017806
Geomyces auratus	KF039895	KF017746	KF017805
