# Supplementary material for: Resource capture and competitive ability of non-pathogenic Pseudogymnoascus spp. and P. destructans, the cause of white-nose syndrome in bats
Source: PLoS One. 2017 Jun 15;12(6):e0178968. doi: 10.1371/journal.pone.0178968 (PMC5472292; doi:10.1371/journal.pone.0178968)
Supplement: S2 Table — See the materials and methods for t-test details, values are rounded to the nearest non-zero integer. (PDF) [file pone.0178968.s004.pdf]

	Pd - 14 days	Pd - 30 days
**SM 7-5-2**	0.005	0.0002
**SM 8-4-5**	0.0003	0.009
**SM 9-3-2**	0.001	0.002
**SM 10-3-2**	0.00008	0.04
**SM 10-3-3**	$8.0E^{-8}$	0.5
**SM 12-9-2**	$8.0E^{-11}$	0.002
